# Long-term care insurance in Italy: medico-legal and socio-economic profiles

**DOI:** 10.3389/fpubh.2024.1405735

**Published:** 2024-07-02

**Authors:** Paolo Bailo, Giuliano Pesel, Filippo Gibelli, Ascanio Sirignano, Giovanna Ricci

**Affiliations:** Section of Legal Medicine, School of Law, University of Camerino, Camerino, Italy

**Keywords:** long-term care insurance (LTCI), non-self-sufficiency insurance, health insurance, long-term care risk protection, non-self-sufficiency risk long-term care (LTC)

## Abstract

Long-term care insurance (LTCI) plays a crucial role in providing substantial aid in non-self-sufficient situations and complementing existing state protection mechanisms. With an aging population and increasing demand for healthcare services LTC policies have become indispensable. While individual LTCI policies face adoption challenges, group insurances offer a more streamlined alternative. However, realizing the full potential of these insurances necessitates targeted legislative intervention to improve accessibility and ensure sustainability. This article explores the evolution of LTCI policies in Italy, offering an overview of the current landscape and highlighting the socio-economic and medico-legal factors shaping the present scenario. By providing this analysis, we seek to offer insights into the dynamic evolution of LTCI policies and the crucial role of legislative measures in enhancing their effectiveness and accessibility.

## 1 Introduction

Insurance policies can provide a range of protections, one of which is long-term care insurance (LTCI) coverage. LTCI is designed to support individuals in case they lose their independence due to illness. There are various insurance policy options available in the market, catering to different needs (1). LTCI originated in the United States and entered the European market in the mid-1980s, reaching Italy as late as 2000 (2).

LTCI policies can address complete loss of self-sufficiency or the inability to perform regular daily activities. They come in individual and group formats and can either be standalone or integrated with other types of insurance. Moreover, they can be categorized as either whole-life or temporary policies. Whole-life policies involve premium payments and underwriting that are limited to a specific age, whereas temporary policies have conditions that must occur during the insurance term or at a predetermined age. The protection provided can involve reimbursing expenses for care or guaranteeing a lifelong annuity in the event of non-self-sufficiency, or it can include offering healthcare services.

The eligibility assessment of LTCI policies is generally based on Activities of Daily Living (ADLs), which include essential self-care activities such as feeding, dressing, washing, using the toilet, walking, and maintaining continence. Nearly all Italian insurance companies use Katz ADL scale to assess daily living activities (3). This scale scores limitations in daily activities, with some insurers grouping ADLs into four main categories: personal hygiene, dressing, walking, and feeding. For these companies, non-self-sufficiency is determined by the total loss of at least three functions. Other insurers adhere closely to Katz original scale, which includes all six ADLs. They assign 5 points for partial loss and 10 points for total loss, with non-self-sufficiency being reached at 40 points (4, 5).

Additionally, there are evaluative scales like Instrumental Activities of Daily Living (IADLs), which also consider cognitive and functional abilities, aspects not covered by the previous scales. IADL scales evaluate more complex tasks like grocery shopping, making phone calls, and managing medication (6).

The need for LTC policies arises from the necessity to ensure financial coverage for non-self-sufficiency, particularly in old age, as seen in many countries, including Italy (7). As the older adult population grows and family care diminishes due to economic constraints, there is a rising demand for healthcare services, highlighting the urgency of securing financial resources.

To better understand the long-term care situation in Italy, the Ministry of Health commissioned a survey in 2023 to analyze the relevant statistics (8). At the beginning of 2023, estimates indicated that Italy had approximately 28 million individuals over the age of 50, with more than half over 65 and 3% over 90. One key metric for assessing long-term care is the number of older adult individuals receiving integrated home care (IHC). IHC comprises a combination of medical, nursing, and rehabilitation treatments, along with social welfare services such as personal hygiene, personal care, and meal assistance, provided directly in the person’s home. Between 2014 and 2022, the number of over-65 s receiving IHC increased from 252,377 to 459,151. Specifically, for those over 75, the numbers rose from 209,781 to 383,250, nearly doubling in just under a decade (8).

Another main indicator is the access to long-term care facilities such as nursing homes, which primarily cater to non-self-sufficient older adult individuals requiring full-time medical, nursing, and rehabilitation care. From 2017 to 2022, the number of over-65 s in nursing homes increased from 296,183 to 362,249. For those over 75, the number grew from 269,392 to 329,545, showing a smaller increase compared to IHC, partly due to the substantial costs associated with these facilities (8).

Relying solely on government welfare and social security is not sufficient in an aging society. Therefore, LTCI policies are crucial to provide stronger household support (9). Current LTC utilization faces challenges such as low demand, insufficient supply, and limited accessibility. Overcoming these barriers and promoting LTCI adoption can be achieved through a group-based approach and prospective legislative initiatives, like tax deductions aimed at alleviating the financial burden and encouraging broader investment in LTCI coverage for future non-self-sufficiency.

This article conducts a comprehensive examination of long-term care insurance in Italy, exploring the current landscape and addressing social and medico-legal challenges tied to its implementation. To provide a comprehensive analysis of LTCI policies, we consulted several sources. These included Italian legislation and the Insurance Code, studies/data by the Italian National Insurance Association (ANIA), individual policies of various companies, and information provided by consumer associations. Beyond presenting the issues, we offer potential solutions, aiming to contribute insights for a more resilient and effective long-term care insurance framework in Italy. In addition, we conducted a search within the offerings of leading insurance companies in Italy to identify the primary LTCI products available at the moment. This involved analyzing the contracts and their respective clauses.

## 2 LTCI in Europe: an overview

Prior to delving into the discussion of LTCI in Italy, it is pertinent to first provide a brief overview of the LTCI landscape across Europe.

The landscape of long-term care insurance in Europe varies significantly across countries, primarily due to differences in institutional frameworks and market development. Overall, relatively few Europeans over the age of 50 hold private long-term care insurances. The main determinants for owning a long-term care policy include education, income, widowhood, good subjective health status, and chronic conditions. Higher levels of education and income are associated with a greater likelihood of purchasing long-term care insurances, while widowed individuals are also more likely to have such policies, possibly due to their increased need for formal care.

France and Israel stand out with well-developed long-term care insurance markets. In Israel, about 60% of the population has some form of private LTCI, either through health plans or commercial insurance. This high penetration rate is driven by three types of LTCI: commercial individual, commercial collective, and collective through health plans. France also has a robust LTCI market, with around 5.5 million people insured, representing approximately 10% of the market. French insurance fills the gap in public care provision, which only partially covers the costs of long-term care (10).

In contrast, other European countries have much lower coverage rates. For instance, Austria, Denmark, the Czech Republic, and Italy have underdeveloped LTCI markets. In Austria, only about 1.45% of the population holds a private LTCI policy, while Denmark has a slightly higher rate at 1.83%. Italy and the Czech Republic also report low coverage rates, with 2.63 and 2.28%, respectively. These low rates are partly due to less developed market structures and fewer insurance options available (10).

Regarding public expenditure, Sweden and the Netherlands spend between 3.5 and 4% of their GDP on public LTCI, while countries like the Czech Republic and Israel spend less than 0.5%. This variation in public spending influences the incentive structures for purchasing private LTCI. In countries with extensive public coverage, such as Sweden and Denmark, there is less need for private insurance. Conversely, in Southern and Eastern Europe, where formal public care is less prevalent, the reliance on informal family care reduces the demand for private LTCI (10).

A small experiment presented in a 2015 study by Bucher-Koenen et al. (10) suggests that if countries such as Austria, Denmark, the Czech Republic and Italy adopted market conditions similar to those in France and Israel, LTCI coverage rates could increase significantly. This suggests that the low LTCI penetration in these countries is more due to supply-side constraints than to differences in socio-demographic factors.

## 3 The Italian landscape: shifting to a collective approach

In Italy, the introduction of LTCI policies into the market around 2000 marked a pivotal response to the challenges posed by an increasingly aging population. At the turn of the century, the impact of an aging demographic was becoming palpable, necessitating proactive measures to accommodate the evolving needs of the population. LTCI policies emerged as a potential solution, aiming to provide comprehensive coverage and support for individuals facing the prospect of aging-related challenges.

The specifics of LTCI coverage are delineated in the Minister of Finance Decree of December 22, 2000, No. 47, titled “Insurance for the risk of non-self-sufficiency in the performance of acts of daily living” (11). Additionally, the Code of Private Insurance (Legislative Decree No. 209/2005) classifies LTCI as “insurance against the risk of non-self-sufficiency” under class IV life insurance. This class encompasses long-term, non-rescindable contracts designed to mitigate the risk of serious disability arising from illness, accident, or longevity (12).

Regarding the activation of insurance coverage, Italian insurance policies are mainly based on the fulfillment of ADLs criteria, requiring the insured person to exhibit impairments in at least three or four ADLs to qualify for coverage. It is noteworthy that the adoption of the IADLs system is infrequent in this context.

Initially, when LTCI policies were introduced to the market, the standard policy commonly featured a basic life annuity starting at age 65. Additionally, it might include an extra annuity if the policyholder faces a decline in self-sufficiency in their daily living activities, with another supplement kicking in after reaching the age of 85. Moreover, the insurance policy contract mandated an annual reassessment of the policyholder’s self-sufficiency status until the age of 85 (13). However, the current trend indicates a shift away from specifying an annuity age in policies, with the focus now solely on activation in the event of a loss of self-sufficiency.

A significant stride in addressing the socioeconomic challenge of caring for dependent individuals was marked by the aforementioned Ministerial Decree number 47 of the Ministry of Finance of 22 December 2000. This decree concretely acknowledged the option of financial “substitution,” specifically through LTCI policies. It delineated key aspects essential for LTCI policies to qualify for tax benefits, focusing on the contract’s duration and the prohibition of withdrawal by the insurer.

The decree also explicitly outlined the essential activities of daily living that determine non-self-sufficiency, equating a person requiring continuous supervision with a non-self-sufficient individual. Nevertheless, the legislative attempt to designate individuals as non-self-sufficient with the loss of even a single act of daily living, even in part, has not been embraced or endorsed by insurance companies. This remains a propositional model. Furthermore, activating the LTCI policy due to a partial loss of an activity appears imprudent, as a mere impairment resulting from a fracture could qualify as a partial loss of functionality. Consequently, the latest proposals in this regard have not garnered support from insurance companies (14).

However, following the initial decree, there was a notable lack of legislative momentum in the subsequent years regarding LTCI policies. These policies faced challenges in gaining widespread acceptance, primarily due to economic constraints related to paying the insurance premium. Moreover, issues in implementing the LTCI service itself emerged, characterized by a shortfall in concrete supply to meet the increasing demand.

Therefore, faced with the prohibitively high costs of individual LTCI policies, innovative insurance products in the LTCI sector evolved to shift the focus from individual coverage to collective bargaining. Specific groups of workers, recognizing the need for protection in the event of non-self-sufficiency, collaborated with employers and trade unions to secure collective LTCI insurances (15, 16).

Presently, the most robust LTCI safeguard lies in collective initiatives led by entities like trade associations or unions. These initiatives compel employers to allocate resources for comprehensive LTCI protection, exemplifying a collective awareness among workers about the shared risk of dependency and the imperative for coverage across the entire group. This collective approach is the linchpin of the sustainability of LTC protection. The sharing of contributions between employers and employees, coupled with a risk calculation that transcends individual assessments, fortifies the resilience of this form of protection. By embracing the collective, the burden of risk is distributed across the entire group membership category, fostering a sense of mutuality where all insured individuals contribute to compensate those affected by adverse events.

The principle of solidarity further underlines this collective effort, as low-risk insureds contribute more than their entitlement to facilitate affordable premiums for high-risk individuals. In this way, collective protection circumvents the anti-selection mechanisms inherent in individual policies. Unlike individual policies where only those at high risk seek LTC protection, collective initiatives prevent insurers from being exposed to an undue number of benefit claims, promoting a more balanced and sustainable LTCI insurance landscape. This approach aimed to relieve the financial burden on individual workers while remaining sustainable for insurance companies. By insuring a well-defined group with a specific risk profile—working-age individuals with a low potential for non-self-sufficiency—the policies became financially viable. Yet, a primary drawback of these LTCI group policies often lies in the loss of coverage for individuals once they retire. Consequently, at the time of greatest need, particularly in old age, individuals may find themselves without the essential coverage they require.

Indeed, group-type LTCI policies can be broadly categorized into two distinct types. The first category imposes a limitation on the renewal of coverage upon reaching retirement age (typically up to a maximum of 70 years) (17). In this scenario, if non-self-sufficiency occurs after retirement, no compensation is provided. Conversely, the second category caters to specific groups of workers (e.g., bankers) (18), allowing the policy activated during working age to extend coverage for non-self-sufficiency throughout the individual’s lifetime, even into advanced age. While the second type is more advantageous, providing continuous coverage, it is exclusive to certain occupational categories and often entails higher costs.

Given the advantages inherent in collective bargaining, integrating public and private intervention emerges as a comprehensive solution, ensuring both sufficient coverage for members and the sustainability of the policy system. Currently, collective bargaining in the LTCI domain finds expression through supplementary pension funds, as regulated by Decree-Law 252/2005 (19), and supplementary health care funds, governed by Presidential Decree No. 917/86 (20), Decree-Law 502/1992 (21), and Decree-Law 229/1999 (22).

These supplementary health funds are open to collaborations between public and private entities, ensuring no risk-selection or discriminatory behavior against specific categories of individuals. Such funds can be either self-managed or entrust the coverage of benefits to both public and private institutions, including insurance companies that may enjoy tax benefits when participating in LTCI protection.

Italy features three major supplementary health funds: the Single National LTC Fund (insurance personal protection), ABI-CASDIC (banking personal protection), and the Mutual Assistance Board for Italian Professionals (EMAPI), covering various professional categories such as physicians, lawyers, chemists, physicists, agronomists, etc. All three entities rely on the ADL method to determine the attainment of non-self-sufficiency and subsequently provide benefits (23).

**Figure 1:**
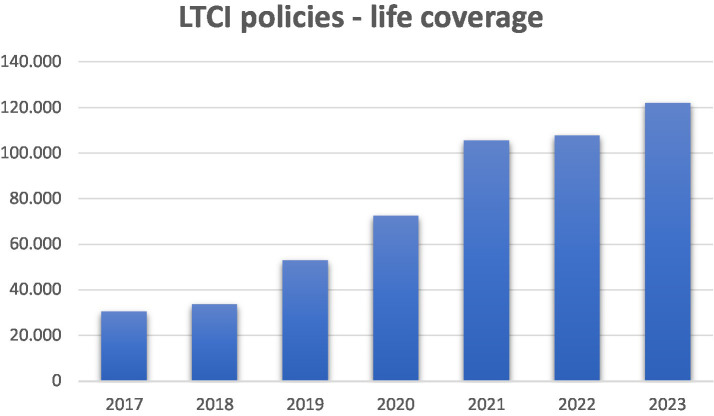
Distribution of LTCI life coverage policies (2017–2023, ANIA data).

To further detail the landscape of LTCI use in Italy, we examined available data to quantify the number of LTCI policies currently in circulation and their distribution between group and individual policies. From newspaper sources (24), the overall estimate is that only 2 percent of the population uses LTCI policies. Reliable data on the number of LTCI policies currently in place are scarce, with available information coming exclusively from ANIA (25). For life coverage LTCI policies between 2017 and 2023, only the total numbers are available, without distinguishing between individual and group policies. Despite a significant increase from 30,000 to over 120,000, these policies still represent only about 0.1–0.2% of all life insurance policies. The data are presented in Figure 1.

**Figure 2:**
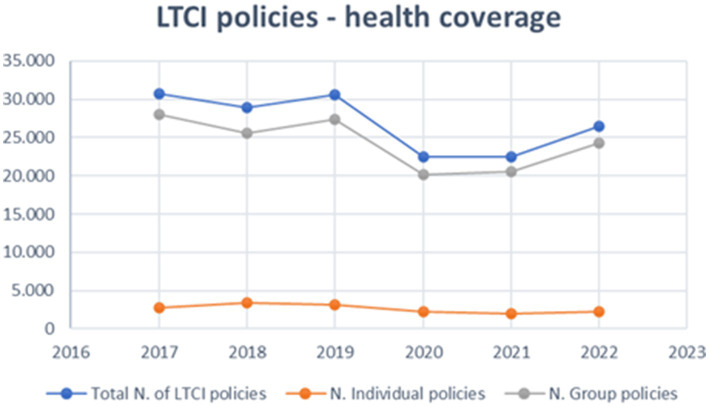
Distribution of LTCI health coverage policies (2017–2022, ANIA data).

Regarding LTCI policies health coverage, the number of insurance policies from 2017 to 2022 ranges between 20,000 and 30,000, with approximately 90% being group policies and the remaining 10% being individual policies. These health insurance policies constitute less than 1% of the total health insurance policies, fluctuating between 0.7 and 1% over the years. A noticeable decline occurred between 2019 and 2020, primarily due to the COVID-19 pandemic, with a modest recovery in 2022. The data are presented in Figure 2. Compared to the significant number of non-sufficient individuals, the uptake of these policies is minimal.

What is the economic impact of non-self-sufficiency on individual households? When a family member is not self-sufficient, it necessitates various management choices. One option is admitting the individual to a nursing home, while another is hiring a full-time caregiver at home. Alternatively, relatives might choose to devote more time to the individual, often reducing their work hours, which has significant economic and psychological repercussions. To provide a clearer picture of the costs involved, several consumer associations and trade unions have estimated the expenses of these services. The average cost of a nursing home is approximately € 1,700–3,500 per month (26), with half of this covered by the state and the remainder, potentially supplemented by municipal contributions, borne by the family. Conversely, employing a regular caregiver with social security contributions costs between € 19,000 and 21,000 annually, not including additional expenses for holiday or sick leave coverage (27,28). Private expenditure on nursing homes and home care is estimated to be around € 33 billion, or 1.7% of gross domestic product. This spending is almost entirely out-of-pocket, imposing a significant financial burden on families. LTCI policies contribute only € 178 million, a mere 0.2% of the total value of life insurance coverage (29).

## 4 Latest initiatives and the current situation

The legislature has taken steps to encourage the adoption of LTCI policies, primarily focusing on employees. In 2017, the legislature intervened in employment contracts to provide tax incentives for individuals opting for LTCI policies (30). Under this initiative, the funds allocated for insurance premiums are exempt from income calculations. This regulatory action aimed to link employment with reasonable protection against future non-self-sufficiency for workers.

Regrettably, this approach does not extend to cover the worker’s family. Furthermore, this strategy has been employed to promote awareness about the risks of non-self-sufficiency and the importance of having LTCI coverage. This shift in awareness has, in turn, influenced workers’ union representatives, who now have a vested interest in enhancing worker protections and facilitating better access to LTC policies.

Concerning the deficiency in services for dependents, the imperative to reshape the Italian welfare system in light of the burgeoning aging population prompted government and parliamentary action as early as 1997. Over the following two decades, various proposals emerged, championed not only by social associations and trade unions but also by a bill that materialized in 2023 as Law 33, officially deliberated by the parliament on March 23, 2023 (31). Law 33/2023 is a delegated law, providing the government with the mandate to establish legislative decrees for the reform of care for dependent older adult individuals. While this law does not directly address LTCI policies, its significance lies in its impact on a fundamental obstacle faced by LTCI policies: the absence of adequate services for dependent individuals.

The most noteworthy aspect of Law 33/2023, particularly relevant to LTCI policies, involves the expansion of services outlined in the enabling act. This expansion translates into increased home, residential, and family services. In terms of home services, models must be established to cater to the specific needs of older adult individuals, considering appropriate durations and hours per day. Territorial health services are to be integrated with municipal services to enhance overall support (29).

For residential services, the focus is on augmenting staffing in older adult residences with more tailored skills to match guest profiles. Introduction of control systems is also proposed to uphold an ideal quality of life. In the realm of family services, particularly in the form of permanent caregivers, there is a call for better definition through specific guidelines outlining required professional skills. Additionally, increased protection for these caregivers is proposed through tax and contribution concessions.

These comprehensive changes collectively contribute to an augmented service landscape. Importantly, policyholders can utilize the reimbursement they receive after losing self-sufficiency to access and benefit from these enhanced services.

The existing landscape of individual LTCI policies in the Italian market is represented in Table 1.

**Table 1:** Italy’s long-term care insurance landscape, focusing on entry age limits and activation criteria: ADL (Activities of Daily Living) or iADL (Instrumental Activities of Daily Living).

Insurance company	Maximum entry age	Activation scale
Generali	74 years and 6 months	ADL
ITAS Assicurazioni	74 years	ADL
UnipolSai	Not provided	ADL
Reale Mutua	70 years	ADL + iADL
Helvetia	65 years	ADL
Allianz	75 years	ADL
Zurich	70 years	ADL
CFAssicurazioni	69 years	ADL
Italiana Assicurazioni	71 years	ADL + iADL
HDI Assicurazioni	70 years	ADL
AXA Assicurazioni	70 years	ADL
Vittoria Assicurazioni	70 years	ADL
Groupama	65 years	ADL
Postevita - Poste Italiane	70 years	ADL
Mediolanum	70 years	ADL
Alleanza Assicurazioni	70 years	ADL

LTCI insurance mainly focuses on life coverage, with little presence in health coverage. In life coverage, the emphasis is on providing an annuity, while in health coverage, LTCI policies aim to cover medical and assistance expenses in case of a loss of self-sufficiency. Activation criteria mainly hinge on basic ADLs, overlooking IADLs. This focus might limit coverage comprehensiveness by ignoring evolving care needs associated with cognitive functions.

Another important aspect is the brief entry period into these policies, contrasting with the overall life expectancy. As indicated in Table 1, individuals can only enroll in the insurance policy up to the age specified by the policy. When workers are young, they often do not consider the need for protection against non-self-sufficiency, or they find LTCI too expensive. However, by the time they reach retirement age, LTCI policies are no longer available. Depending on the insurance company, no one over the age of 65–75 can apply for LTCI. Therefore, when a person reaches the terminal stages of life while still self-sufficient—thanks to increased life expectancy—they cannot secure LTCI if they have not already done so. This exclusionary factor significantly contributes to the low adoption rates of LTCI policies. A more inclusive approach to policy entry timelines could address this issue, ensuring that a diverse range of individuals, including those at later stages of life, can benefit from LTCI coverage.

To promote wider adoption of LTCI policies, innovative ideas have been proposed, such as integrating LTCI contribution into mandatory auto liability insurance. This proposal aims to address the substantial loss of self-sufficiency resulting from traffic accidents. By distributing this cost across society, the premium per insured individual would be substantially reduced, promoting financial accessibility and underscoring the collective responsibility of society in safeguarding the well-being and long-term care needs of its members (15).

## 5 Medico-legal considerations

LTCI policies raise challenging medical-legal issues. Foremost among these is the methodology employed by insurance companies to assess an individual’s loss of self-sufficiency. In Italy, the national insurance association (ANIA) defines this loss in terms of basic activities of daily living (31, 32). However, this definition is overly restrictive, offering a one-dimensional perspective that fails to consider an individual’s unique capabilities within their social and relational contexts. A more accurate interpretation of loss of self-sufficiency should encompass not only fundamental biological functions but also encompass complex actions that manifest at the social and relational levels. In essence, the scales for ADLs should evolve to include communication-sensory skills, comprehension skills and overall quality of life, or alternatively these scales should be abandoned, and new criteria should be formulated to better define the state of non-self-sufficiency.

Currently, LTCI policies often employ criteria that do not consider the social and relational aspects of an individual, relying instead on outdated parameters that inadequately assess an insured person’s loss of independence. For example, a severe psychiatric condition may allow for the performance of elementary activities but still result in a significant loss of autonomy. The German and French ADL scales address this issue by considering variables such as the time caregivers spend providing assistance (33, 34).

In addition, legally significant exclusions in insurance coverage include conventional ones (e.g., criminal activities, hazardous sports) and a proposed addition of “blameworthy conduct” forgoing entitlement to annuity for those disregarding medical advice. This raises complexities in determining expected actions from the insured regarding medical advice and resolving disputes from conflicting medical opinions. Enforcing compliance with medical recommendations may conflict with established regulations (8). Under Italian law, patients have the right to make their own health care decisions, and doctors must respect their autonomy. When an insurance company turns medical advice into an imposition, it violates this legislation. For example, patients may choose not to follow dietary advice or weight reduction plans intended to lower the risk of ischemic events, and this choice must be respected.

The last critical issue involves the interaction between LTCI and liability insurance, specifically in cases where a loss of self-sufficiency is not due to natural aging but rather caused by the actions of others, such as in a traffic accident. A question arises: should the compensation from liability insurance be added to the benefits of the LTCI policy? In a strict sense, the LTCI policy should not be cumulative when it comes to loss of self-sufficiency resulting from the actions of third parties. In such cases, it is these third parties who are responsible for compensation, which excludes the need for LTCI policy indemnity. Furthermore, government welfare insurance interventions should also be considered when assessing the economic burden, thus reducing the liability of the insurance company providing the LTCI policy. Currently, there is no specific legislation or regulation in the Italian context to address this issue. Consequently, LTCI policies continue to be cumulative in cases of loss of self-sufficiency caused by the actions of third parties (10).

## 6 Strategies for policy implementation

In order to maximize the effectiveness of LTCI policies, an intervention should be envisaged that integrates legislative interventions, market development, public-private partnerships and awareness-raising campaigns. Such an intervention should in our view include the following initiatives:

- Legislative intervention: introducing targeted legislation to improve the accessibility and sustainability of LTCI. This should include tax incentives for both individuals and employers who opt for LTCI. Legislation should also mandate the inclusion of LTCI in employment contracts, ensuring coverage continues post-retirement.
- Promotion of group policies: encouraging the adoption of group LTCI policies through collective bargaining agreements. Policymakers should facilitate collaborations between employers, trade unions, and insurance providers to offer LTCI as part of employee benefit packages. This approach leverages the lower cost and broader coverage potential of group policies.
- Public-private partnerships: establishing partnerships between public welfare systems and private insurance companies to provide comprehensive LTCI solutions. These partnerships can help bridge the gap in coverage and ensure that services are available to all segments of the population, including low-income individuals.
- Enhancement of services for dependents: expanding home, residential, and family care services as outlined in recent legislative efforts. This includes increasing staffing in older adult residences, integrating territorial health services with municipal services, and providing tax and contribution concessions for permanent caregivers.
- Awareness and education campaigns: launching nationwide campaigns to raise awareness about the importance of LTCI and the risks of non-self-sufficiency. These campaigns should target both the general public and specific groups such as young workers and retirees, emphasizing the financial and emotional benefits of having LTCI.
- Development of comprehensive assessment criteria: revising the criteria for assessing non-self-sufficiency to include social and relational aspects, not just basic ADLs. New scales that take into account cognitive function and overall quality of life should be implemented, ensuring a more accurate determination of eligibility for LTCI benefits.
- Incorporation of LTCI into auto liability insurance: integrating LTCI contributions into mandatory auto liability insurance to cover the substantial loss of self-sufficiency resulting from traffic accidents. This approach distributes the cost across society, reducing individual premiums and promoting accessibility.
- Continuous monitoring and evaluation: establishing a monitoring body to oversee the implementation and effectiveness of LTCI policies. This body should regularly assess market penetration, the quality of services provided, and the financial sustainability of LTCI products, making adjustments as necessary.
- Support for caregivers: providing specific guidelines and increased protections for caregivers through professional development programs and financial support. This ensures caregivers are well-equipped to provide high-quality care and reduces the financial burden on families.
- Expansion of eligibility and enrollment periods: extending the eligibility age for enrolling in LTCI policies to include older adults who may not have considered the need for such coverage when younger. This inclusive approach ensures a broader segment of the population can benefit from LTCI.

## 7 Conclusion

LTCI policies represent a valuable instrument to enhance support in scenarios of non-self-sufficiency, serving as a complementary layer to state protection. Regrettably, the adoption of individual policies has been limited, yet the potential for efficiency lies in the utilization of group policies. To foster a wider adoption of this beneficial product, we contend that legislative intervention is imperative. Such intervention has the potential not only to render this kind of product more economically accessible but, more importantly, to initiate a self-sustaining cycle of increased awareness, adoption, and overall sustainability. A strategic legislative approach can play a pivotal role in optimizing the appeal and viability of LTC policies, thereby ensuring enhanced assistance for those in need and fortifying the overall welfare system.

## 8 Study limitations

The present study has several limitations.

Firstly, the article primarily focuses on the Italian context, which may limit the generalizability of its findings to other countries with different healthcare systems and socio-economic conditions. Additionally, the data sources used in the study, such as those from the Italian National Insurance Association (ANIA) and various consumer associations, may have inherent biases or limitations in accuracy and comprehensiveness. The reliance on specific legislative texts and insurance policies also means that the study might not fully capture recent or unpublished changes in the market or regulatory environment.

Another significant limitation is the potential for variability in the implementation and interpretation of ADLs and IADLs criteria across different insurers, which could affect the comparability of results. Furthermore, the analysis of LTCI policies does not deeply explore the individual experiences and outcomes of policyholders, which could provide valuable insights into the real-world effectiveness of these policies.

Finally, while the article suggests legislative interventions to improve LTCI policy accessibility and sustainability, it does not provide empirical evidence on the potential impacts of such interventions, making these recommendations more speculative than substantiated by data.

## Data availability statement

The original contributions presented in the study are included in the article. Further inquiries can be directed to the corresponding author.

## Author contributions

PB: Conceptualization, Methodology, Writing – original draft, Writing – review & editing. GP: Conceptualization, Methodology, Writing – review & editing. FG: Writing – original draft, Writing – review & editing. AS: Project administration, Resources, Supervision, Writing – review & editing. GR: Methodology, Project administration, Resources, Supervision, Writing – original draft, Writing – review & editing.
